# Utilization of modern menstrual methods and related unmet needs among college going women in Coimbatore district: a descriptive cross-sectional study

**DOI:** 10.1186/s12905-024-02915-5

**Published:** 2024-01-30

**Authors:** Thavansree Durairaj, Periasamy Aparnavi, Seetharaman Narayanan, Sushmitha Mahantshetti, Srihari Dhandapani, Jeevithan Shanmugam, Ramesh Rathinamoorthy, Mohan Kumar

**Affiliations:** 1 KMCH Institute of Health Sciences and Research, Coimbatore, Tamil Nadu India; 2 Department of Community Medicine, KMCH Institute of Health Sciences and Research, Coimbatore, Tamil Nadu India

**Keywords:** Modern hygiene management, Menstrual cups, Tampons, Adolescents, India, Unmet needs

## Abstract

**Objectives:**

The primary objectives were to determine the proportion of modern menstrual method (MMM) users among college going women in Coimbatore district, Tamil Nadu; and to estimate the unmet needs associated with use of MMMs in comparison with other menstrual hygiene methods (MHMs). We also assessed the factors that determine MMM use among college going women.

**Methods:**

This was a descriptive cross-sectional study conducted among college going women in Coimbatore district, Tamil Nadu, India between October 2022 and January 2023 using a purpose predesigned, pretested, semi-structured proforma that included validated Menstrual Practice Needs Scale (MPNS-36).

**Results:**

Only 1.4% of the study participants used MMMs – menstrual cups (1.3%) and tampons (0.1%). Sanitary pads were the most common MHM of choice (96.3%); of which majority (98.6%) used disposable pads and more than half (50.4%) used non-biodegradable pads. Importantly, one in six (16.5%) were not aware of nature of sanitary pads (biodegradable or nonbiodegradable) used. The unmet needs associated with MMMs (menstrual cups and tampons) were significantly lower than that for other MHMs (including sanitary pads), in particular, the unmet material and home environment needs, unmet material reliability concerns, unmet reuse needs and unmet reuse insecurity. However, we found no significant difference between MMMs, sanitary pads and other MHMs in terms of unmet transport, college environment, change and disposal insecurity needs. The significant predictors of use of MMMs were age (more than 21 years of age), residence (urban), type of stay (off campus including home), socioeconomic status (upper), fathers’ and mothers’ education (high school and above), and presence of personal income. Discussions with friends (or peers) both before and after menarche regarding menstruation resulted in higher adoption of modern menstrual methods.

**Conclusion:**

MMMs provided comparative advantage with lesser unmet needs for material reliability and reuse insecurity concerns, particularly in home environment. However, none of the MHMs fulfilled the user expectations for transport and disposal insecurity concerns, particularly outdoors.

## Introduction

Menstruation is a normal physiological process; a part of reproductive events in primate females [1]. There are over 355 million menstruating girls and women in India; millions of them still facing significant barriers to a comfortable and dignified experience with menstrual hygiene management [2]. With the understanding that the pattern and pace of adoption of different menstrual hygiene methods differ greatly between different societies, in a low- and middle-income setting like India, the preference of menstrual hygiene method is based on cultural acceptability, economic status, and availability in the local market; more than personal choice [3, 4]. For instance, though tampons and menstrual cups have become the method of choice in western societies decades ago, they are yet to find a foothold in Indian communities [5]. Tampons and menstrual cups (so called modern menstrual methods (MMM)) are not the preferred choices, given the apprehension among women with products involving vaginal insertion, and potential loss of virginity. In most Indian states, the use of sanitary napkins is considered “conventional” and is still the most widely used method [6]. The utilization of disposable sanitary pads can present a secure option for females if used with regularity and proper hygiene [7]. However, the management and disposal of these products have become a growing concern. Improper disposal methods include discarding menstrual waste in public spaces, urban sewage systems, landfills, rural areas, and water bodies without following any standard procedures [8]. Consequently, what starts as an individual problem escalates into a broader social issue.

The National Family Health Survey – 5 (NFHS-5) defines hygienic methods of protection during the menstrual period as use of locally prepared napkins, sanitary napkins, menstrual cups, or tampons during their menstrual period by women between 15 and 24 years of age. The data highlighted that 78.0% of women in this age group used a hygienic method of menstrual protection [9]. However, adequate menstrual hygiene involves having access to sanitary materials that are clean and can be changed privately whenever necessary. It also includes access to soap and water for washing and a proper place for disposing of used sanitary materials or washing them, particularly when reusable pads are used [10]. In India, there is a notable scarcity of data concerning the unmet needs of women related to available menstrual hygiene methods, hindering comprehensive understanding of the challenges and gaps in menstrual hygiene practices among women in the country. Inadequate menstrual hygiene has been linked to infections (approximately 70% of the reproductive tract infections in Indian women are due to poor menstrual hygiene) and a diminished quality of life concerning health [11–14]. In urban adolescents in the United States, negative experiences related to menstruation have been associated with higher rates of school absenteeism and missing out on activities [15]. 

Against this background, the primary objectives of the present study were to determine the proportion of modern menstrual method users among college going women in Coimbatore district, Tamil Nadu, and to estimate the unmet needs associated with use of modern menstrual methods in comparison with other menstrual hygiene methods. We also assessed the factors that determine modern menstrual method use among college going women.

## Methods

This was a descriptive cross-sectional study conducted among college going women in Coimbatore district, Tamil Nadu, India between October 2022 and January 2023. The study enrolled all women studying in colleges of Coimbatore district. However, we excluded participants not willing to provide digital informed consent. A line list of universities and colleges in Coimbatore district was prepared by referring to the Public Utilities directory available at the District Collectorate, Coimbatore district, Government of Tamil Nadu; disaggregated by type of institution (university or college), location (urban or rural), type of ownership (government or private), and courses offered (medicine, allied health sciences, arts and science, and commerce). We randomly (simple random sampling technique) choose six colleges (because of feasibility concerns only six colleges were chosen) – one university and five colleges; two from rural and four from urban; three arts and science colleges, one medical, allied health science and commerce college each. Each college was visited twice. During the first visit, permission was sought from respective heads of the institution, study rationale was explained, and consent/assent forms were circulated to all eligible participants (complete enumeration). In the second visit, a purpose predesigned, pretested, semi-structured proforma in Google Forms platform (https://forms.gle/AwUa3wmhS68DwT177) that included socio-demographic characteristics, choice of menstrual hygiene methods and validated Menstrual Practice Needs Scale (MPNS-36) was administered [16]. Considering the proportion of modern menstrual method users among adolescent girls to be 2.3%, the minimum estimated sample size was 3106 (with sample size formula for estimating a single proportion) for estimating the expected proportion with 20% precision relative to the expected proportion and 95% confidence.

We defined the choice of menstrual hygiene method as the most preferred method or that method currently used (last menstrual period) [9]. Menstrual cups and tampons were considered modern menstrual methods. We estimated the unmet needs associated with use of modern menstrual methods in comparison with other menstrual hygiene methods using Menstrual Practice Needs Scale (MPNS-36). MPNS-36 is a set of 36 self-report questions quantitatively capturing the women’s perceptions of comfort, satisfaction, adequacy, reliability as well as worries and concerns during the last menstrual period (four domains namely material and home environment needs, transport and college environment needs, material reliability concerns, change and disposal insecurity for disposable methods; additionally, two domains namely reuse needs and reuse insecurity for reusable methods). It measures the extent to which respondents’ menstrual management practices and environments were perceived to meet their needs during their last period. The scale has demonstrated acceptable reliability and validity; Cronbach’s alpha value of 0.78, test-retest reliability coefficient or intraclass correlation coefficient (ICC) ranging between 0.66 and 0.69, and content validity index of 0.89 [16, 17]. 

The data obtained using Google Forms was exported in Microsoft Excel format and analysed using Statistical Package for the Social Sciences (SPSS) v27. Descriptive analysis was presented using numbers and percentages for categorical variables and mean (standard deviation) for continuous variables with appropriate graphs. To test for association between MPNS-36 domain scores and choice of menstrual hygiene method we used one-way analysis of variance (ANOVA) assuming equal variance with Bonferroni correction to adjust for probability values because of the increased risk of a type I error when making multiple statistical tests. This was adjusted for factors significantly associated with modern menstrual method use among college going women. To test for association between independent study variables and choice of menstrual hygiene methods we used Chi square test (two sided). Statistical significance was considered at *p* < 0.05.

## Results

The present study included a total of 3144 college going women in Coimbatore district, Tamil Nadu. The mean (SD) age of the study population was 19.34 years (1.83), with a median (IQR) of 19.00 years (18.00 to 20.00) and ranging between 16 and 46 years.

### Proportion of modern menstrual method users

**Fig. 1:**
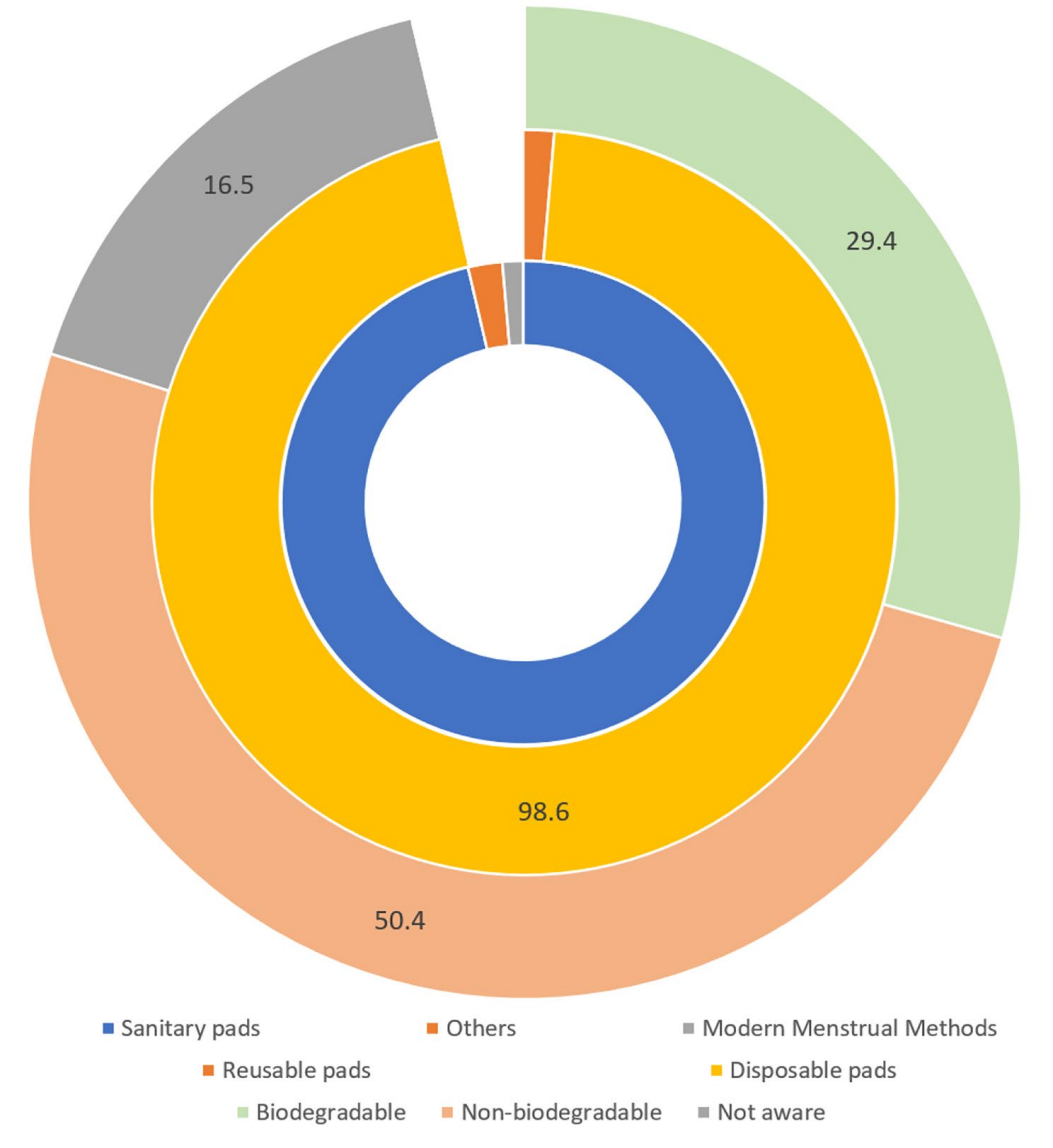
Distribution of choice of menstrual hygiene method

Only 1.4% of the study participants used modern menstrual methods – menstrual cups (1.3%) and tampons (0.1%). Majority (96.3%) of the study participants used sanitary pads during their last menstrual period (current choice) – of which 98.6% used disposable and 1.4% used reusable pads. We also noted that 50.4% participants used non-biodegradable pads, 29.4% used biodegradable pads and 16.5% were not aware of biodegradability (Fig. 1).

The other menstrual hygiene methods practised by the participants (2.3%) in the present study were cotton wool, natural materials (including use of mud, leaves, grass), normal underwear alone, strips of sari, towel, or other cloth and toilet paper or tissues.

### Unmet needs associated with use of modern menstrual methods

The mean MPNS scores were significantly lower for sanitary pad users (Mean 1.89, SD 0.44) and users of other menstrual hygiene methods (Mean 1.75, SD 0.44) in comparison with MMM users (Mean 2.10, SD 0.52). In other words, the unmet needs associated with MMMs were significantly lower than sanitary pads and other menstrual hygiene methods (*p* < 0.05). It was found that the unmet material and home environment needs associated with MMMs (Mean 2.32, SD 0.69) was significantly lower in comparison with use of sanitary pads (Mean 2.06, SD 0.66) and other menstrual hygiene methods (Mean 1.68, SD 0.85) (*p* < 0.05). Similarly, the unmet material reliability concerns were significantly lower among MMM users (Mean 2.10, SD 0.73) in comparison with sanitary pad users (Mean 1.72, SD 0.78). However, we found no significant difference between MMMs, sanitary pads and other menstrual hygiene methods in terms of unmet transport, college environment, change and disposal insecurity needs (*p* > 0.05) (Table 1a).

**Table 1a:** Unmet needs associated with menstrual hygiene methods

	MMM *N* = 43	Sanitary pads *N* = 3029	Others *N* = 72	Total *N* = 3144
	Mean (SD)	Mean (SD)	Mean (SD)	Mean (SD)
MPNS scores – Total	2.10 (0.52)	1.89 (0.44)	1.75 (0.44)	1.89 (0.44)
	Ref	0.005*	< 0.001*	
Material and home environment needs	2.32 (0.69)	2.06 (0.66)	1.68 (0.85)	2.05 (0.66)
	Ref	0.032*	< 0.001*	
Transport and college environment needs	1.78 (0.84)	1.75 (0.72)	1.56 (0.81)	1.74 (0.73)
	Ref	1.000	0.372	
Material reliability concerns	2.10 (0.73)	1.72 (0.78)	1.90 (0.87)	1.73 (0.79)
	Ref	0.005*	0.571	
Change and disposal insecurity	2.16 (0.81)	2.03 (0.74)	1.87 (0.85)	2.03 (0.74)
	Ref	0.797	0.136	
**For those using reusable materials (** ***N*** ** = 446)**				
Reuse needs	2.26 (0.67)	1.66 (0.80)	1.68 (0.77)	1.71 (0.81)
	Ref	< 0.001*	0.044*	
Reuse insecurity	2.16 (0.85)	1.64 (0.82)	1.88 (0.56)	1.69 (0.83)
	Ref	0.001*	0.777	

*Statistically significant at *p* < 0.05

**Table 1b:** Unmet needs associated with menstrual hygiene methods

		MMM *N* = 43	Sanitary pads *N* = 3029	Others *N* = 72	Total *N* = 3144	*p* value
		n (%)	n (%)	n (%)	n (%)	
Unmet needs (Overall)	Present	18 (41.9)	1901 (62.8)	56 (77.8)	1975 (62.8)	0.001*
	Absent	25 (58.1)	1128 (37.2)	16 (22.2)	1169 (37.2)	
Unmet material and home environment needs	Present	13 (30.2)	1374 (45.4)	50 (69.4)	1437 (45.7)	< 0.001*
	Absent	30 (69.8)	1655 (54.6)	22 (30.6)	1707 (54.3)	
Unmet transport and college environment needs	Present	30 (69.8)	2117 (69.9)	54 (75.0)	2201 (70.0)	0.646
	Absent	13 (30.2)	912 (30.1)	18 (25.0)	943 (30.0)	
Unmet material reliability concerns	Present	18 (41.9)	2202 (72.7)	43 (59.7)	2263 (72.0)	< 0.001*
	Absent	25 (58.1)	827 (27.3)	29 (40.3)	881 (28.0)	
Unmet change and disposal insecurity	Present	17 (39.5)	1464 (48.3)	41 (56.9)	1522 (48.4)	0.177
	Absent	26 (60.5)	1565 (51.7)	31 (43.1)	1622 (51.6)	
**For those using reusable materials (** ***N*** ** = 446)**						
Unmet reuse needs	Present	14 (41.2)	289 (73.2)	12 (70.6)	315 (70.6)	< 0.001*
	Absent	20 (58.8)	106 (26.8)	5 (29.4)	131 (29.4)	
Unmet reuse insecurity	Present	15 (44.1)	308 (78.0)	14 (82.4)	337 (75.6)	< 0.001*
	Absent	19 (55.9)	87 (22.0)	3 (17.6)	109 (24.4)	

*Statistically significant at *p* < 0.05

For participants using reusable methods, it was found that the unmet reuse needs associated with MMMs (Mean 2.26, SD 0.67) was significantly lower in comparison with sanitary pads (Mean 1.66, SD 0.80) and other menstrual hygiene methods (Mean 1.68, SD 0.77). Similarly, the unmet reuse insecurity needs were significantly lower among MMM users in comparison with sanitary pad users (*p* < 0.05).

Unmet needs associated with MMMs was significantly lower (41.9%) than sanitary pads (62.8%) and other MHMs (77.8%) (Table 1b). The estimated overall unmet material and home environment needs were 45.7%, unmet transport and college environment needs were 70.0%, unmet material reliability concerns were 72.0%, unmet change and disposal insecurity needs were 48.4%, unmet reuse needs were 70.6%, and unmet reuse insecurity needs were 75.6%. A significantly lower proportion of MMM users (30.2%) had unmet material and home environment needs in comparison with sanitary pads (45.4%) and others menstrual hygiene methods users (69.4%). Similarly, MMM users had a significantly lower unmet material reliability concerns, unmet reuse needs, and unmet reuse insecurity (*p* < 0.05). No method performed better in terms of transport, college environment needs, change and disposal insecurity (*p* > 0.05) – unmet transport and college environment needs ranged between 69.8% for MMM users, 69.9% for sanitary pad users and 75.0% for users of other menstrual hygiene methods; unmet change and disposal insecurity needs ranged between 39.5% for MMM users, 48.3% for sanitary pad users and 56.9% for users of other menstrual hygiene methods.

### Factors associated with modern menstrual method use

The results of the present study showed that a significantly (*p* < 0.05) higher proportion of MMM users were more than 21 years of age (23.3%), from urban areas (current residence, 76.7%), with off campus type of stay (including home, stay with relatives, hostels, as paying guests, and similar, 86.1%), upper socioeconomic status (69.8%), with fathers’ and mothers education high school and above (83.7% and 88.4% respectively), and presence of personal income source (14.0%). However, marital status, hometown, and freedom to manage day-to-day expenses were not statistically associated with use of modern menstrual methods in the present study (*p* > 0.05) (Table 2a).

**Table 2a:** Factors associated with choice of menstrual hygiene methods

		MMM *N* = 43	Sanitary pads *N* = 3029	Others *N* = 72	Total *N* = 3144	*p* value
		n (%)	n (%)	n (%)	n (%)	
Age (in years)	≤ 21	33 (76.7)	2738 (90.4)	67 (93.1)	2838 (90.3)	0.008*
	> 21	10 (23.3)	291 (9.6)	5 (6.9)	306 (9.7)	
Marital status	Unmarried	41 (95.3)	2979 (98.3)	70 (97.2)	3090 (98.3)	0.253
	Married/ Separated/ Divorced	2 (4.7)	50 (1.7)	2 (2.8)	54 (1.7)	
Current residence	Rural	10 (23.3)	921 (30.4)	32 (44.4)	963 (30.6)	0.022*
	Urban	33 (76.7)	2108 (69.4)	40 (55.6)	2181 (69.4)	
Hometown	Rural	17 (39.5)	1341 (44.3)	39 (54.2)	1397 (44.4)	0.201
	Urban	26 (60.5)	1688 (55.7)	33 (45.8)	1747 (55.7)	
Type of current stay	Home	23 (53.5)	1997 (65.9)	53 (73.6)	2073 (65.9)	0.001*
	In campus hostel	6 (14.0)	628 (20.7)	7 (9.7)	641 (20.4)	
	Off campus^@^	14 (32.6)	404 (13.3)	12 (16.7)	430 (13.7)	
Socioeconomic status	Lower and middle	13 (30.2)	1858 (61.3)	50 (69.4)	1921 (61.1)	< 0.001*
	Upper	30 (69.8)	1171 (38.7)	22 (30.6)	1223 (38.9)	
Fathers’ education	Illiterate and/or up to middle school	7 (16.3)	748 (24.7)	27 (37.5)	782 (24.9)	0.019*
	High school and above	36 (83.7)	2281 (75.3)	45 (62.5)	2362 (75.1)	
Mothers’ education	Illiterate and/or up to middle school	5 (11.6)	694 (22.9)	24 (33.3)	723 (23.0)	0.024*
	High school and above	38 (88.4)	2335 (77.1)	48 (66.7)	2421 (77.0)	
Freedom to manage day-to-day expenses	Absent	11 (25.6)	1167 (38.5)	31 (43.1)	1209 (38.5)	0.160
	Present	32 (74.4)	1862 (61.5)	41 (56.9)	1935 (61.5)	
Personal source of income	Absent	37 (86.0)	2883 (95.2)	67 (93.1)	2987 (95.0)	0.018*
	Present	6 (14.0)	146 (4.8)	5 (6.9)	157 (5.0)	

*Statistically significant at *p* < 0.05

^@^Off campus stay with relatives, hostels, as paying guests, and similar.

**Table 2b:** Factors associated with choice of menstrual hygiene methods

Discussion about menstruation		MMM *N* = 43	Sanitary pads *N* = 3029	Others *N* = 72	Total *N* = 3144	*p* value
		n (%)	n (%)	n (%)	n (%)	
Before menarche, mother/sister/any family member	No	11 (25.6)	1221 (40.3)	26 (36.1)	1258 (40.0)	0.117
	Yes	32 (74.4)	1808 (59.7)	46 (63.9)	1886 (60.0)	
Before menarche, friend(s)	No	12 (27.9)	1408 (46.5)	42 (58.3)	1462 (46.5)	0.007*
	Yes	31 (72.1)	1621 (53.5)	30 (41.7)	1682 (53.5)	
After menarche, mother/sister/any family member	No	2 (4.7)	188 (6.2)	7 (9.7)	197 (6.3)	0.433
	Yes	41 (95.3)	2841 (93.8)	65 (90.3)	2947 (93.7)	
After menarche, friend(s)	No	4 (9.3)	260 (8.6)	15 (20.8)	279 (8.9)	0.001*
	Yes	39 (90.7)	2769 (91.4)	57 (79.2)	2865 (91.1)	

*Statistically significant at *p* < 0.05

We also assessed whether discussions with family members (mother and/or sister) and friends about menstruation would predict the choice the modern menstrual methods. It was found that discussions with friends both before (72.1%) and after (90.7%) menarche regarding menstruation resulted in higher adoption of modern menstrual methods (*p* < 0.05). However, such an association was not found to be statistically significant for discussion with family members (mothers and/or sisters) (Table 2b).

## Discussion

This descriptive cross-sectional study found that only 1.4% college going women in Coimbatore district, Tamil Nadu used modern menstrual methods (menstrual cups and tampons). Sanitary pads were the most common menstrual hygiene method of choice (96.3%); of which majority (98.6%) used disposable pads and more than half (50.4%) used non-biodegradable pads. Importantly, one in six (16.5%) were not aware of nature of sanitary pads (biodegradable or nonbiodegradable) used. Nearly two third (62.8%) college women had unmet needs with current choice of menstrual hygiene methods. The unmet needs associated with modern menstrual methods were significantly lower than that for other menstrual hygiene methods (including sanitary pads), in particular, the unmet material and home environment needs, unmet material reliability concerns, unmet reuse needs and unmet reuse insecurity. However, we found no significant difference between MMMs, sanitary pads and other menstrual hygiene methods in terms of unmet transport, college environment, change and disposal insecurity needs. The significant predictors of use of modern menstrual methods were age (more than 21 years of age), residence (urban), type of stay (off campus including home, stay with relatives, hostels, as paying guests, and similar), socioeconomic status (upper), fathers’ and mothers’ education (high school and above), and source of personal income. Our results also showed that discussions with friends (or peers) both before and after menarche regarding menstruation resulted in higher adoption of modern menstrual methods. However, such an association was not found to be statistically significant for discussion with family members (mothers and/or sisters).

The adoption of modern menstrual methods, such as menstrual cups and tampons, was quite low, with only 1.4% of participants reporting their usage. This finding corroborates with existing literature evidence. The dominance of sanitary pads as the primary menstrual hygiene product is consistent with previous studies that have highlighted the popularity of pads among Indian women due to their ease of use, availability, and affordability [18, 19]. The National Family Health Survey – 5 (NFHS-5, 2019-21) documented that the percent distribution of women 15 to 24 years of age who have ever menstruated to be 0.3% for menstrual cups and 1.7% for tampons. In a recent cross-sectional study reported from Gujarat, 2.27% college students used menstrual cups (0.70%) and tampons (1.57%); whereas use of sanitary pads was the most common (96.06%) [20]. van Eijk AM et al. (2016) conducted a systematic review to summarize the status of menstrual hygiene management (MHM) among adolescent girls in India. The study found that only two studies reported use of tampons (one from urban Tamil Nadu and the other from urban Karnataka) and none reported menstrual cups [21]. Insertable menstrual products such as menstrual cups and tampons are rarely used, although there are local manufacturers. Modern menstrual methods may face cultural barriers and misconceptions (including concerns about virginity, insertion of foreign objects into the body, societal expectations to conform to traditional practices, stigma attached to menstruation and menstrual blood or menstruating women considered impure limits MHM related discussion) [22], making them less popular choices in this region. To add to this, lack of awareness, easy accessibility, availability (including availability of appropriate size), affordability, and supportive environment (including physical, social environment and support system for guidance) are the barriers to adoption of modern menstrual methods. Multiple studies described user familiarisation with the menstrual cup over time (learning curve of 2–5 months), with practice, peer support, and training being key to success [23–25]. 

Menstrual cups minimises the economic burden (given that one cup can last up to 10 years) and menstrual waste compared to the use of sanitary pads; [26] they were safe, convenient (1.4 DALYs averted, 95% CI -4.3 to 3.1), and acceptable for girls and/or women in studies reported from Nepal [27], Kenya [25, 28], Canada [29], South Africa [30], , and India [31]. Similar findings have been reported from other low- and middle-income countries including Africa [32, 33]. Though Oster E et al. reported that menstrual cups have the benefit of being easy to clean requiring less water [27], literature specifies the need for adequate water supply along with clean washroom facility (essential and not desirable) for menstrual cup use. The motivation to use menstrual cups is ingrained with the detrimental experiences of using sanitary pad (such as developing rashes, bad odour, discomfort, and disposal) [34]. 

The magnitude of unmet needs signify the gap in meeting menstrual health requirements of young women in the region warranting attention. Comparatively, MMMs were associated with lesser unmet needs. Literature refers to MMMs as better menstrual hygiene solutions [35] than can hold more menstrual fluid, reducing the frequency of changes (can hold 10 to 38 mL of blood; should be emptied every 4 to 12 h, depending on menstrual flow and type of cup) and offering increased convenience and freedom of movement [30, 36]. The lower level of unmet needs in MMMs, particularly regarding material reliability, reuse concerns, and home environment needs, highlights their potential to address some of the challenges faced by traditional methods like sanitary pads. The reusable nature of menstrual cups makes them cost-effective and environmentally friendly. Babagoli MS et al. estimated the costs of menstrual cups to be $3,270 per year for 1000 girls, compared with $24,000 for sanitary pads. The menstrual cup intervention was cost-effective in improving health outcomes ($2,300/DALY averted) [37]. 

It is important to note that modern menstrual methods may not completely eliminate all unmet needs. For example, we observed no significant difference between MMMs, sanitary pads, and other menstrual hygiene methods in terms of unmet transport, college environment, change, and disposal insecurity needs. These aspects can be influenced by factors such as access to clean and private restroom facilities (proper sanitation facilities) at colleges (or other educational institutions and public places), availability of menstrual products at educational institutions, availability of disposal options, and cultural perceptions of menstruation [36, 38]. Briefly, modern menstrual methods, menstrual cups (made of medical-grade silicone, rubber, latex, or elastomer) in particular, provided better material reliability, and reuse security [39]. However, it was associated with lesser unmet needs only in home environment and none of the menstrual hygiene methods performed better in external environment (transport or place of education). Addressing these broader contextual issues is crucial to ensuring comprehensive menstrual hygiene management.

The high prevalence of unmet needs with traditional menstrual hygiene methods, especially sanitary pads, could be attributed to various factors. Firstly, the adverse effects (including rashes, reproductive tract infection, vaginal infections, cervical cancer, urinary tract infection, hepatitis B, and different types of yeast infections) associated with use of sanitary pads may impact the quality of life of women [7, 13]. Secondly, the cost constraints might limit the accessibility of high-quality disposable pads for some individuals [40]. Additionally, concerns about the environmental impact of disposable pads may lead to insecurities about their usage and disposal [41]. A recent estimate showed that one billion pads per month (or 12 billion pads per year) are used and being disposed of in India – 33.0% buried, 28.0% along with routine waste, 28.0% in open, and 28% burnt in open. Use of superabsorbent polymers, nonbiodegradable plastic, glue etc. does not allow decomposition of disposed pads for a minimum of 500 to 800 years. Additionally, it causes long term deterioration of water and soil quality. Blood soiled menstrual absorbents are best culture medium for disease causing pathogens as well [8]. 

Similar to the findings of this study, in many societies, the menstrual practices may evolve with age, influenced by factors such as cultural norms, peer influence, education, access to information, and financial independence. Increased awareness through educational institutions (including environmental awareness), internet access, and peer discussions (including positive testimonials) may positively influence the adoption of menstrual cups and tampons among older college-going women [42]. Financial autonomy may enable them to explore and afford modern menstrual products that can be relatively more expensive upfront but offer long-term cost benefits. In contrast, younger girls might depend on their parents or guardians for menstrual products, limiting their choices to more affordable options like traditional pads. Comfort and familiarity with one’s body play an important role in adopting different menstrual methods. Younger girls might be more hesitant to try something new and may prefer sticking to the menstrual hygiene practices they (or the immediate family) are already accustomed to. As girls grow older, they might develop a deeper understanding of their bodies and become more open to exploring alternative menstrual products.

Urban residence typically offers greater access to information, products (attributable to distribution networks (including online shopping) and market availability), healthcare services (facilities and specialists), and a supportive home environment, all of which can influence the uptake of modern menstrual methods. Similar to the type of residence, socioeconomic status and parents’ education can influence various factors, such as awareness, affordability, cultural beliefs, and access to information, all of which may shape individual preferences for menstrual hygiene products. Individuals from higher SES backgrounds or with educated parents may have better exposure to health-related knowledge, including information on menstrual hygiene products [43]. The higher upfront (or capital) costs of menstrual cups (and the availability of alternate methods with relatively low capital costs) can create disparities in the adoption of modern menstrual methods based on economic status [7, 37]. Families with higher education levels may be more open to discussing menstruation openly and may be less influenced by traditional taboos; and may be proactive in seeking information from healthcare providers, leading to better-informed decisions (improved health seeking behaviour). In contrast, families with lower education levels might adhere more strictly to traditional practices and may be hesitant to consider or try modern menstrual methods. Ultimately, efforts to promote menstrual hygiene and MMMs should be tailored to address the specific needs and challenges faced by individuals from different socioeconomic backgrounds, with a focus on promoting equitable access to safe menstrual health products and education.

The present study highlights the importance of peer influence and social networks in shaping menstrual hygiene practices, particularly when it comes to adopting modern menstrual methods like menstrual cups and tampons. The influence of friends and peers on adolescent behaviours and decisions is well-documented [44]. In the context of menstrual hygiene, discussions with friends before and after menarche can play a crucial role in normalizing the use of modern menstrual methods. As young girls begin to menstruate and navigate their menstrual experiences, the support and experiences shared by friends can influence their attitudes towards and acceptance of modern menstrual products. Conversely, the statistical insignificance shown by discussions with family members, such as mothers and sisters, could be attributed to cultural taboos and discomfort around discussing menstruation within families. In some communities, particularly in LMICs, menstruation remains a sensitive and private topic, making it less likely for girls to openly discuss alternative menstrual products with their family members.

The present study is not without limitations. Firstly, the present study quantified the unmet needs associated with menstrual hygiene methods. Though a validated questionnaire that assessed multiple dimensions (MPNS-36) was used to capture unmet needs, an additional qualitative method (either in-depth interviews or focus group discussions) would have been useful. Secondly, we could establish association and not causation between menstrual hygiene method and unmet needs. However, we adjusted the results for possible predictors of choice of menstrual hygiene methods, so that the unmet needs could be attributed to the method itself.

To conclude, the adoption of modern menstrual methods, such as menstrual cups and tampons, was quite low in the present study. The study also highlights an alarming lack of awareness regarding the biodegradability of sanitary pads, potentially contributing to environmental concerns associated with menstrual waste management. Unmet needs related to menstrual hygiene were prevalent among a substantial proportion of college girls and women, particularly in terms of current menstrual hygiene choices. MMMs provided comparative advantage with lesser unmet needs for material reliability and reuse insecurity concerns, particularly in home environment. However, none of the menstrual hygiene methods fulfilled the user expectations for transport and disposal insecurity concerns, particularly outdoors. The uptake of MMMs is much higher with peer discussions. Policymakers, educators, and healthcare providers should collaborate to create a supportive environment that encourages open discussions about menstruation and menstrual hygiene. Empowering women with accurate information and access to a variety of menstrual products can play a crucial role in improving overall menstrual health and well-being in the Coimbatore district and beyond.

## Data Availability

The datasets used and/or analysed during the current study available from the corresponding author on reasonable request.
